# Association between physical performance or parameters and sarcopenia

**DOI:** 10.1097/JS9.0000000000000653

**Published:** 2023-08-11

**Authors:** Dongxu Chen, Qiyu He, Zhimin Tan, Shijian Feng

**Affiliations:** a Department of Anesthesiology, West China Second Hospital of Sichuan University; b The Key Laboratory of Birth Defects and Related Diseases of Women and Children, Ministry of Education, Sichuan University; c Department of Urology; d Department of Anesthesiology, West China Hospital of Sichuan University, Chengdu, Sichuan Province, People’s Republic of China

*Dear Editor*,

Sarcopenia, a condition characterized by the degenerative loss of muscle mass, strength, and function, has been found to affect a significant number of patients undergoing surgery, with prevalence rates ranging from 30 to 74%^1^. Surgical patients, particularly those undergoing total knee or hip replacement, often experience muscle loss. Several studies have already suggested that impaired muscle mass can greatly impact postoperative outcomes^2^. As proposed in a recent article published in *International Journal of Surgery*, muscle mass, as a reflection thereof, can be objectively measured^3^. However, the role of east–west differences remains unclear, as well as evidence regarding various parameters among the Chinese population is still limited. The primary objective of this prospective observational study is to investigate the correlation between a patient’s physical performance or parameters and the presence of sarcopenia.

This study conforms to the ethical guidelines of the Declaration of Helsinki and was approved by the local institutional review board (approval No. 2019/169). Prior to their participation in this study, all patients signed informed consent. A total of 118 consecutive patients [median age, 65 (interquartile range, 55–70) years; 77 females (65.3%)] undergoing orthopedic surgery were included. We determined the status of sarcopenia based on a set of well-established scales (Chinese version) collected through face-to-face interviews at baseline. Specifically, the presence of sarcopenia was evaluated by the 5-item SARC-F questionnaire (score range 0–10, including strength, assistance with walking, rising from a chair, climbing stairs, and falls), which a score equal to or greater than 4 is predictive of sarcopenia^4^. In addition to demographic characteristics, various physical parameters were measured for each patient, including bilateral mid-upper arm and calf circumference, grip strength, and a 6-meter walking speed test. Furthermore, the patient’s functional status (i.e. Barthel Index) and nutritional assessment [Nutritional risk screening (NRS 2002)] were also taken into consideration (Table 1).

**Table 1:** Physical examination tests, stratified by the presence of sarcopenia (yes or no).

Characteristic	Total	Sarcopenia	No sarcopenia	*P*
Number of participants	118	28	90	
Mid-upper arm circumference, cm				
Left				
Median (IQR)	28.2 (26.5–29.5)	28.9 (27.6–31.1)	28.0 (26.2–29.4)	0.069
Mean (SD)	28.4 (3.0)	29.4 (2.4)	28.2 (3.1)	0.084
Right				
Median (IQR)	28.3 (26.5–30.0)	29.1 (27.9–30.9)	28.2 (26.3–30.0)	0.140
Mean (SD)	28.6 (3.3)	29.4 (2.4)	28.4 (3.4)	0.146
Calf circumference, cm				
Left				
Median (IQR)	33.45 (31.43–36.00)	33.65 (32.28–34.93)	33.30 (31.05–36.00)	0.783
Mean (SD)	33.51 (3.21)	33.53 (3.27)	33.50 (3.21)	0.964
Right				
Median (IQR)	33.4 (31.0–35.8)	33.8 (31.6–35.4)	33.2 (31.0–36.0)	0.837
Mean (SD)	33.5 (3.3)	33.4 (3.2)	33.5 (3.3)	0.912
Grip strength, kg				
Left				
Median (IQR)	23 (19–28)	21 (18–24)	23 (20–29)	0.018
Mean (SD)	24 (7)	21 (5)	25 (7)	0.003
Right				
Median (IQR)	23 (21–28)	21 (20–26)	24 (21–29)	0.013
Mean (SD)	25 (7)	22 (4)	26 (7)	0.002
Barthel Index				
Median (IQR)	90 (85–95)	83 (80–85)	90 (90–95)	<0.001
Mean (SD)	90 (7)	82 (5)	92 (6)	<0.001
Nutritional risk screening (NRS 2002)				
Median (IQR)	1.00 (1.00–2.00)	1.00 (1.00–2.00)	1.00 (1.00–2.00)	0.645
Mean (SD)	1.50 (0.77)	1.50 (0.64)	1.52 (0.81)	0.430
6-meter walking speed test, m/s				
Median (IQR)	1.61 (1.40–1.84)	0.85 (0.69–1.24)	1.70 (1.54–1.94)	<0.001
Mean (SD)	1.59 (0.49)	0.99 (0.41)	1.77 (0.35)	<0.001

The values are reported as the median (interquartile range) and mean (standard deviation) for continuous variables.

We, in total, identified 28 (23.7%) sarcopenia cases. By the presence of sarcopenia, we found patients with sarcopenia were more likely to be female (82.1 vs. 60.0%; eTable 1, Supplemental Digital Content 1, http://links.lww.com/JS9/A878). Furthermore, our analysis revealed that sarcopenia was strongly associated with decreased grip strength in both arms, as indicated by the median grip strength measurements (left: 21 vs. 23 kg; right: 21 vs. 24 kg; refer to Fig. 1A and eTable 2, http://links.lww.com/JS9/A878). In addition, we observed a noticeable disparity in the results of the 6-meter walking speed test. Patients with sarcopenia exhibited significantly slower walking speed (median: 0.85) compared to those without the condition (median: 1.70). These findings indicate that sarcopenia not only affects physical performance but also impacts functional abilities, as supported by the lower median Barthel Index scores in sarcopenic patients (83 vs. 90). Comparable findings were derived from the correlation analysis (Fig. 1B), wherein sarcopenia demonstrated an inverse relationship with grip strength (*r*=−0.31 to −0.32), function scores (*r*=−0.77), and walking speed (*r*=−0.32).

**Figure 1:**
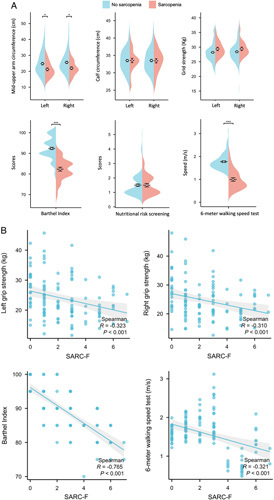
(A) The physical performance status in surgical patients. (B) Correlation analysis between grip strength, Barthel Index, 6-meter walking speed, and the presence of sarcopenia. SARC-F, strength, assistance with walking, rising from a chair, climbing stairs, and falls questionnaire.

This study has several limitations, primarily due to its single-center design and small sample size. Additionally, the changes in sarcopenia status during the follow-up after surgery were not assessed. In clinical practice, magnetic resonance imaging, computed tomography, dual-energy X-ray absorptiometry, and bioelectrical impedance analysis are commonly used to diagnose decreased muscle mass. However, these methods can be expensive or involve exposure to radiation^5^. Therefore, there is a need for inexpensive and safe independent methods for the early diagnosis of sarcopenia. Our study findings indicate that grip strength, functional status scores, and the 6-meter walking speed test can serve as simple proxy screening tools for sarcopenia. Based on our results, we recommend conducting physical performance assessments during the initial clinical encounter to better categorize patients and minimize the risk of unfavorable outcomes .

## Ethical approval

Ethical approval was obtained from the Institutional Review Board (approval No. 2013-169), and informed consent was obtained from all enrolled patients.

## Sources of funding

None.

## Author contribution

D.C. and S.F.: conceptualization; Q.H. and Z.T.: data acquisition; Q.H: statistical analysis; Z.T.: creation of the table and the figure; D.C. and S.F.: writing– review and editing and supervision. All the authors approved the final manuscript as submitted and agree to be accountable for all aspects of the work.

## Conflicts of interest disclosure

The authors declare that they have no conflicts of interest.

## Research registration unique identifying number (UIN)

1. Name of the registry: Relationship between BCAAs metabolism and sarcopenia.
2. Unique identifying number or registration ID: ChiCTR2100049413.
3. Hyperlink to your specific registration (must be publicly accessible and will be checked): https://www.chictr.org.cn/showproj.html?proj=131130&u_atoken=6f7a70e1-5033-44fa-9fee824f02a6e35f&u_asession=01BzgCqA9qi92XPkNhxhhYhwE23tB1p 6US0YgJZzi5bNnq8OH4k4TaYpF52GUogmKPX0KNBwm7Lovlpxj d_P_q4JsKWYrT3W_NKPr8w6oU7K_T4QtGpOEmNNHO_xTQHG 5wfLQ4WAzKMpeQ5gBYVoJommBkFo3NEHBv0PZUm6pbxQU& u_asig=05_XdN3PuBKh5kzXoHuxuBP_GOwMio_c79QD8I6Z8hK6 0j5Q4bu7S7Lw7krdIvWu6Hgg8Ru3QjvJmhCHVZVKN8a7RoBNlM Y9dBGaj-Tn54RoiD4Y-P46j1xI3l2n0UyelIaSvFCbHorbQTgXH9ZE5VlRCk-wb3qr1nbOw_8TnChP9JS7q8ZD7Xtz2Lyb0kmuyAKRFSVJkkdwVUnyHAIJzeJV2mLsd0oDBubneRmDLrcV6 BPOz54AISrxQnoPkMsQLCDZcaGZC2eDyMNrbIBtJ3h9VXwMyh6PgyDIVSG1WMZgfMJ_Pdjt3R8WobthEF32TG907D0gWFGobKr9p4gvTTMc6iYuX5RMVcy682gl8JCCeFa690Qf8eHusMufmWspDxyAEEo4kbsryBKb9Q&u_aref=9x4cPPrmb9zKVw3YxyVjO5rkvd8%3D.

## Guarantor

All authors.

## Data availability statement

The data presented in this study are available on request from the corresponding author.

## Supplementary Material
