# Supplementary material for: Association between physical performance or parameters and sarcopenia
Source: Int J Surg. 2023 Aug 11;109(11):3720–3. doi: 10.1097/JS9.0000000000000653 (PMC10651246; doi:10.1097/JS9.0000000000000653)
Supplement: Supplementary file 1 [file js9-109-3720-s001.docx]

**Appendix eTable 1. Baseline and surgery characteristics, stratiﬁed by the presence of** **sarcopenia (yes or no).**

| **Characteristic** | **Total** | **Sarcopenia** | **No sarcopenia** | **P-value** |
| --- | --- | --- | --- | --- |
| No. of participants | 118 | 28 | 90 |  |
| Age, y |  |  |  |  |
| Median (IQR) | 65 (55, 70) | 67 (58, 71) | 63 (54, 70) | 0.143 |
| Mean (SQ) | 62 (11) | 66 (8) | 61 (12) | 0.054 |
| Female sex, No. (%) | 77 (65.3) | 23 (82.1) | 54 (60.0) | 0.032 |
| Height, cm |  |  |  |  |
| Median (IQR) | 158 (154, 163) | 156 (152, 159) | 160 (155, 165) | 0.015 |
| Mean (SQ) | 160 (13) | 156 (6) | 161 (15) | 0.010 |
| Weight, Kg |  |  |  |  |
| Median (IQR) | 61 (55, 68) | 63 (60, 65) | 60 (54, 68) | 0.227 |
| Mean (SQ) | 62 (10) | 63 (8) | 62 (11) | 0.454 |
| Smoking status, No. (%) |  |  |  | 0.149 |
| Never | 89 (75.4) | 25 (89.0) | 64 (71.1) |  |
| Ever | 7 (5.9) | - | 7 (7.8) |  |
| Current | 22 (18.7) | 3 (11.0) | 19 (21.1) |  |
| Drinking status, No. (%) |  |  |  | 0.159 |
| Never | 88 (74.6) | 24 (85.7) | 64 (71.1) |  |
| Ever | 10 (8.5) | - | 10 (11.1) |  |
| Current | 20 (16.9) | 4 (14.3) | 16 (17.8) |  |
| Aged-adjusted charlson comorbidity |  |  |  |  |
| Median (IQR) | 3.00 (2.00, 3.75) | 3.00 (2.00, 3.25) | 3.00 (2.00, 3.75) | 0.207 |
| Mean (SQ) | 2.69 (1.17) | 2.96 (0.88) | 2.60 (1.23) | 0.090 |
| Diabetes mellitus, n (%) | 12 (10.2) | 4 (14.3) | 8 (8.9) | 0.475 |
| Hypertension, n (%) | 42 (35.6) | 11 (39.3) | 31 (34.4) | 0.640 |
| Coronary heart disease, n (%) | 3 (2.5) | 1 (3.6) | 2 (2.2) | 0.560 |
| Chronic obstructive pulmonary disease, n (%) | 4 (3.4) | 2 (7.1) | 2 (2.2) | 0.238 |
| Osteoporosis, n (%) | 42 (35.6) | 10 (35.7) | 32 (35.6) | 0.988 |
| American Society of Anesthesiologists classification, n (%) |  |  |  | 0.108 |
| II | 80 (67.8) | 16 (57.1) | 64 (71.1) |  |
| III | 32 (27.1) | 11 (39.3) | 21 (23.3) |  |
| Unknown | 6 (5.1) | 1 (3.6) | 5 (0.6) |  |
| Type of surgery, n (%) |  |  |  | 0.067 |
| Total hip replacement | 25 (21.2) | 2 (7.1) | 23 (25.6) |  |
| Total knee replacement | 39 (33.1) | 13 (46.4) | 26 (28.9) |  |
| Spinal-related | 54 (45.7) | 13 (46.4) | 41 (45.6) |  |
| SARC-F scores |  |  |  |  |
| Median (IQR) | 2.00 (1.00, 3.00) | 5.00 (5.00, 6.00) | 1.00 (0.25, 3.00) | <0.001 |
| Mean (SQ) | 2.34 (1.94) | 5.21 (0.88) | 1.44 (1.14) | <0.001 |

The values are reported as the median (interquartile range) for continuous variables and n (%) for categorical variables.

Abbreviation: SARC-F, Strength, assistance with walking, rising from a chair, climbing stairs, and falls questionnaire.

**eTable 2.** **P****hysical examination tests, stratiﬁed by the presence of sarcopenia (yes or no).**

| **Characteristic** | **Total** | **Sarcopenia** | **No sarcopenia** | **P-value** |
| --- | --- | --- | --- | --- |
| No. of participants | 118 | 28 | 90 |  |
| **Mid-upper arm circumference, cm** |  |  |  |  |
| **Left** |  |  |  |  |
| Median (IQR) | 28.2 (26.5, 29.5) | 28.9 (27.6, 31.1) | 28.0 (26.2, 29.4) | 0.069 |
| Mean (SD) | 28.4 (3.0) | 29.4 (2.4) | 28.2 (3.1) | 0.084 |
| **Right** |  |  |  |  |
| Median (IQR) | 28.3 (26.5, 30.0) | 29.1 (27.9, 30.9) | 28.2 (26.3, 30.0) | 0.140 |
| Mean (SD) | 28.6 (3.3) | 29.4 (2.4) | 28.4 (3.4) | 0.146 |
| **Calf circumference, cm** |  |  |  |  |
| **Left** |  |  |  |  |
| Median (IQR) | 33.45 (31.43, 36.00) | 33.65 (32.28, 34.93) | 33.30 (31.05, 36.00) | 0.783 |
| Mean (SD) | 33.51 (3.21) | 33.53 (3.27) | 33.50 (3.21) | 0.964 |
| **Right** |  |  |  |  |
| Median (IQR) | 33.4 (31.0, 35.8) | 33.8 (31.6, 35.4) | 33.2 (31.0, 36.0) | 0.837 |
| Mean (SD) | 33.5 (3.3) | 33.4 (3.2) | 33.5 (3.3) | 0.912 |
| **Grip strength, Kg** |  |  |  |  |
| **Left** |  |  |  |  |
| Median (IQR) | 23 (19, 28) | 21 (18, 24) | 23 (20, 29) | 0.018 |
| Mean (SD) | 24 (7) | 21 (5) | 25 (7) | 0.003 |
| **Right** |  |  |  |  |
| Median (IQR) | 23 (21, 28) | 21 (20, 26) | 24 (21, 29) | 0.013 |
| Mean (SD) | 25 (7) | 22 (4) | 26 (7) | 0.002 |
| **Barthel Index** |  |  |  |  |
| Median (IQR) | 90 (85, 95) | 83 (80, 85) | 90 (90, 95) | <0.001 |
| Mean (SD) | 90 (7) | 82 (5) | 92 (6) | <0.001 |
| **Nutritional risk screening**  **(NRS 2002)** |  |  |  |  |
| Median (IQR) | 1.00 (1.00, 2.00) | 1.00 (1.00, 2.00) | 1.00 (1.00, 2.00) | 0.645 |
| Mean (SD) | 1.50 (0.77) | 1.50 (0.64) | 1.52 (0.81) | 0.430 |
| **6-meter walking speed test, m/s** |  |  |  |  |
| Median (IQR) | 1.61 (1.40, 1.84) | 0.85 (0.69, 1.24) | 1.70 (1.54, 1.94) | <0.001 |
| Mean (SD) | 1.59 (0.49) | 0.99 (0.41) | 1.77 (0.35) | <0.001 |

The values are reported as the median (interquartile range) and mean (standard deviation) for continuous variables.
